# Long-term follow-up of endoscopic papillectomy and the value of preventive pancreatic stent placement (with videos)

**DOI:** 10.1093/gastro/goad050

**Published:** 2023-10-18

**Authors:** Yuling Wang, Xiaoqing Zhang, Zhenhua Yang, Teng Wang, Dongqing Zhu, Jie Gao, Ping-Ping Zhang, Peiqin Wang, Xingang Shi

**Affiliations:** Department of Gastroenterology, Changhai Hospital, Naval Military Medical University, Shanghai, P. R. China; Department of Emergency, Changhai Hospital, Naval Military Medical University, Shanghai, P. R. China; Department of Nephrology, Gongli Hospital, Pudong New Area, Shanghai, P. R. China; Department of Gastroenterology, Changhai Hospital, Naval Military Medical University, Shanghai, P. R. China; Department of Radiology, Changzheng Hospital, Naval Military Medical University, Shanghai, P. R. China; Department of Gastroenterology, Changhai Hospital, Naval Military Medical University, Shanghai, P. R. China; Department of Gastroenterology, Changhai Hospital, Naval Military Medical University, Shanghai, P. R. China; Department of Gastroenterology, Changzheng Hospital, Naval Military Medical University, Shanghai, P. R. China; Department of Gastroenterology, Changhai Hospital, Naval Military Medical University, Shanghai, P. R. China

**Keywords:** endoscopic resection, pancreatic duct stent, pancreatitis, adenoma, duodenal papilla

## Abstract

**Background:**

Early-stage ampullary adenomas have only been reported in a small case series on endoscopic management. Hence, this study aimed to evaluate the long-term outcomes of early ampullary adenoma with endoscopic management and identify the risk factors for acute pancreatitis after endoscopic papillectomy (EP).

**Methods:**

In this study, 115 patients who underwent EP at Changhai Hospital (Shanghai, China) between January 2012 and December 2018 were retrospectively analysed. Endoscopy was performed at 1, 3, 6, and 12 months after EP. Data were statistically analysed using the *t*-test or the Mann–Whitney *U* test.

**Results:**

A total of 107 patients were included in this study and the follow-up period was 75 ± 43 months. The average age of the 107 patients was 54.6 years and the average tumor size was 17 mm. The average age of the patients (53.7 ± 10.7 years vs 55.2 ± 10.5 years, *P *=* *0.482), minimum tumor size (13 vs 19 mm, *P *=* *0.063), and complete resection rate (84.78% vs 85.25%, *P *=* *0.947) did not differ significantly between the stent placement and non-stent placement groups. Post-EP acute pancreatitis rates in the non-stent placement and stent placement groups were 11.48% and 4.35%, respectively. The risk of post-EP acute pancreatitis was significantly associated with the preoperative carcinoembryonic antigen level in univariate analysis, but not in multivariate analysis. The risk of post-EP acute pancreatitis was not significantly associated with the placement of the pancreatic stent in either univariate or multivariate analysis. Moreover, delayed proximal pancreatic duct stenosis was not noted in either group during long-term follow-up.

**Conclusions:**

EP is a satisfactory option for treating adenomas of the ampulla of the duodenum.

## Introduction

Adenoma of the ampulla of Vater is rare and the incidence rate is only 0.04%–0.12% [1]. Advances in endoscopic technology have led to a significant increase in the diagnosis of duodenal papillary tumors, with benign lesions being the most commonly encountered type. Therapies for duodenal papillary tumors include pancreaticoduodenectomy, local tumor resection, and robotic-assisted pancreatoduodenectomy [2–4]. Unfortunately, pancreaticoduodenectomy is associated with a mortality rate of 2%–10%, which results in decreased patient satisfaction with the curative treatment [5, 6]. In such cases, endoscopic papillectomy (EP) is a more effective and less invasive management option for ampullary adenomas [7, 8].

EP is currently considered a safe and effective intervention for the resection of adenomatous lesions. Although the mortality rate associated with EP is low (0%–1%), the recurrence rate of EP can be as high as 33% [9]. Nonetheless, the results from long-term follow-up tend to vary among healthcare centers.

Similarly to those receiving medical intervention, patients undergoing EP experience several complications, such as pancreatitis, bleeding, perforation, and cholangitis, which should be considered; of these, post-operative pancreatitis is quite common. A small randomized–controlled trial involving 19 patients identified that the incidence of pancreatitis was significantly higher in the group without stent implantation than in the group with stent implantation (33% vs 0%, *P *=* *0.02) [10]. Proximal pancreatic duct stenosis has also been reported as a prevalent complication of endoscopic ampullectomy, although there is a paucity of relevant literature.

This retrospective study aimed to assess the long-term outcomes of patients undergoing EP for adenomatous ampullary lesions. Furthermore, this study examined the risk factors for post-EP acute pancreatitis and addressed the value of preventive pancreatic duct stenting in the endoscopic resection of duodenal ampullary tumors via long-term follow-up of patients who had undergone EP.

## Patients and methods

### Ethics approval and consent to participate

The study was conducted in accordance with the ethical guidelines of the Declaration of Helsinki. As this was a retrospective study, the requirement for ethical approval was waived by the Ethics Committee of Changhai Hospital (Shanghai, China).

### Patient selection and follow-up

The clinical data of 115 patients who had undergone EP at Changhai Hospital between January 2012 and December 2018 were retrospectively collected.

Inclusion criteria were as follows: (i) histopathological confirmation of adenoma via a biopsy, (ii) no malignancy based on endoscopic appearance, and (iii) no biliary duct or pancreatic duct invasion. Exclusion criteria were as follows: (i) histopathological findings of adenocarcinoma, (ii) a tendency for hemorrhage, or (iii) a follow-up period of <6 months.

Endoscopy was performed at 1, 3, 6, and 12 months after the completion of endoscopic papillectomy (EP) and annually thereafter. During follow-up, if a new neoplastic lesion of <1 cm in diameter was identified at or near the site of the original lesion, it was regarded as a residual tumor. If a neoplastic lesion was noted within 6 months of ESD, local recurrence was considered.

### Resection technique

All papillectomies were performed by an experienced endoscopist using a therapeutic duodenoscope (Olympus TJF260; Olympus Optical, Tokyo, Japan), with the patients receiving sedation anesthesia. Saline mixed with epinephrine (1:10,000) and indigo carmine was injected submucosally to locate the benign tumor. Papillectomy was performed using an Elton injection needle and an oval rigid diathermic loop (2.5 cm × 5.5 cm, Cook Medical). Throughout the procedure, endoscopic hemostasis was successfully achieved using adrenaline injection, hemoclips, and plasma Argon coagulation. As the insertion of a pancreatic duct stent (short, 5F, single-pigtail, Cook Medical) is not a routine step after EP, it was performed only in certain patients. Therefore, the study population was grouped according to the placement of the pancreatic duct stent (i.e. the non-stent placement and stent placement groups; endoscopic surgery videos in Supplementary Videos 1 and 2).

EP was deemed successful if the following criteria were met: (i) no duodenal submucosal invasion and (ii) no recurrence of the lesion during the long-term follow-up. If these criteria were not fulfilled, the procedure was considered a failure.

### Outcome measurement

We evaluated the long-term outcomes of patients who had undergone EP, including their survival rate and recurrence rate, through follow-up assessments. We examined the risk factors for post-EP in acute pancreatitis and the probability of pancreatitis and pancreatic duct stenosis after EP with or without pancreatic duct stenting.

### Statistical analysis

Both continuous variables (such as age) and categorical variables (such as sex) were measured. Continuous variables are presented as the mean and standard deviation or the median and ranges, and the independent sample *t*-test or Mann–Whitney *U* test was used to compare the data between the groups. Categorical variables are presented as relative frequencies and percentages, and the chi-squared test or Fisher’s exact test was used to compare these data between the groups. All *P*-values of <0.05 were considered statistically significant. The statistical software SPSS version 23.0 was used for the analysis.

## Results

### Patient characteristics

Among 115 patients analysed, 3 were excluded owing to biliary or pancreatic duct invasion, 3 were excluded owing to loss of follow-up, and 2 were excluded owing to neuroendocrine tumors; finally, 107 patients were included in the study. All of the 107 patients had undergone EP. Their average age was 54.6 years and the average tumor size was 17 mm (4–50 mm). Most patients experienced abdominal pain and discomfort. In total, 31 cases of adverse events were noted, including bleeding (*n *=* *12), jaundice/biliary obstruction (*n *=* *7), pancreatitis (*n *=* *9), and perforation (*n *=* *3). The percentages of en bloc and complete resections were 81.31% and 85.05%, respectively. Of the 107 patients, 90 (84.11%) had low-grade dysplasia, 12 (11.21%) had high-grade dysplasia, and 5 (4.67%) had carcinoma (Table 1). Furthermore, 13 patients experienced residual or recurrent tumors (Supplementary Table 1).

**Table 1.** Baseline of the patient characteristics

Characteristic	Total (*n *=* *107)
Male gender, *n* (%)	71 (66.36)
Age, years	54.6 ± 10.6
Symptoms, *n* (%)	72 (67.29)
Abdominal pain	57
Abdominal discomfort	13
Maximum diameter, median (range), mm	17 (4–50)
Adverse events, total, *n* (%)	31 (28.97)
Delayed bleeding	12
Jaundice/biliary obstruction	7
Pancreatitis	9
Perforation	3
Death	0
Follow-up, median (range), months	50.7 (6–193)
Post-operative recurrence, *n* (%)	13 (12.15)
Resection, *n* (%)	
En bloc	87 (81.31)
Piecemeal	20 (18.69)
Complete resection	91 (85.05)
Late pancreatic stenosis	0
Final histologic findings, *n* (%)	
Low-grade dysplasia	90 (84.11)
High-grade dysplasia	12 (11.21)
Carcinoma *in situ*	5 (4.67)

### Patient characteristics in the stent placement and non-stent placement groups

The stent placement group comprised 32 men and 14 women, with an average age of 53.7 ± 10.7 years and an average tumor size of 13 mm, whereas the non-stent placement group comprised 39 men and 22 women, with an average age of 55.2 ± 10.5 years and an average tumor size of 19 mm. Five cases of bleeding, three cases of jaundice/biliary obstruction, two cases of pancreatitis, and two cases of perforation were noted in the stent placement group. On the contrary, seven cases of bleeding, four cases of jaundice/biliary obstruction, seven cases of pancreatitis, and one case of perforation were observed in the non-stent placement group. Moreover, two patients developed moderate post-operative pancreatitis. No cases of delayed distal pancreatic duct stenosis were seen during the follow-up in either group (Table 2).

**Table 2.** Patient characteristics in the stent placement and non-stent placement groups

Characteristic	Stent placement group	Non-stent placement group	*P*-value
Male patients, *n*	32/14	39/22	0.542
Age, mean ± SD, years	53.7 ± 10.7	55.2 ± 10.5	0.482
Average diameter, median (range), mm	13 (4–30)	19 (4–50)	0.069
Patients with biliary stent, M/F, *n*	1/16	3/4	0.059
**Adverse events, *n***			
Delayed bleeding	5	7	0.922
Jaundice/biliary obstruction	3	4	0.698
Acute pancreatitis	2	7	0.335
Mild	2	7	
Severe	0	0	
Perforation	2	1	0.804
Post-operative recurrence, *n*	4	9	0.342
Canceration and surgical operation, *n*	1	0	
**Resection, *n* (%)**			
En bloc	41 (89.13)	46 (75.41)	0.071
Complete resection	39 (84.78)	52 (85.25)	0.947
**Final histologic findings, *n* (%)**			
Low-grade dysplasia	37 (80.43)	53 (86.87)	0.366
High-grade dysplasia	5 (10.87)	7 (11.48)	0.922
Carcinoma	4 (8.69)	1 (1.64)	0.212

### Risk factors for post-EP acute pancreatitis in the stent placement and non-stent placement groups

In our study, the percentage of pancreatitis after EP was 8.41%. The risk of post-EP acute pancreatitis was significantly associated with preoperative CEA level in univariate analysis, but not in multivariate analysis. The risk of post-EP acute pancreatitis was not significantly associated with age, sex, tumor size, preoperative symptoms or histology in either univariate or multivariate analysis (Table 3).

**Table 3.** Risk factors and development of post-procedure acute pancreatitis in 107 patients who underwent endoscopic papillectomy

Factor	Pancreatitis		Univariate analysis	Multivariate analysis	
	Yes	No	*P*-value	95% CI	*P*-value
Age, mean ± SD, years	52.2 ± 11.3	54.7 ± 10.5	0.493	0.847–1.011	0.084
Gender, M/F, *n*	7/2	46/52	0.154	0.038–2.077	0.213
Tumor size, mean ± SD, mm	16 ± 9	16 ± 10	0.990	0.789–1.173	0.702
Final histologic findings, *n*			0.121	0.021–1.234	0.079
LGD	8	82			
HGD	–	12			
Ca	1	4			
Pancreatic stenting, *n*			0.192	0.072–3.517	0.487
No	7	54			
Yes	2	44			
Preoperative symptoms, *n*			0.837	0.257–5.866	0.797
No	4	46			
Yes	5	52			
Preoperative CEA, *n*			0.033	0.191–46.749	0.435
High	1	4			
Low	8	94			
Preoperative CA199, *n*			0.458	0.006–7.116	0.377
High	1	5			
Low	8	93			

SD, standard deviation; LGD, low-grade dysplasia; HGD, high-grade dysplasia; Ca, carcinoma; CI, confidence interval.

### Kaplan–Meier estimates of disease-free survival rates between the pancreatic stent placement and non-stent placement groups

**Figure 1.**
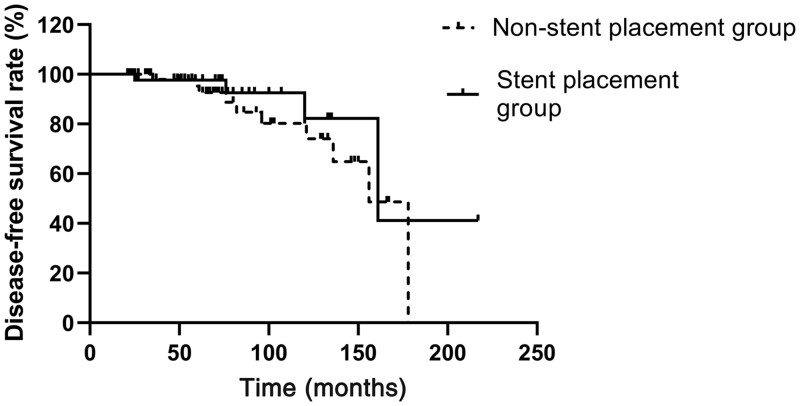
Kaplan–Meier estimates of disease-free survival rates between the pancreatic stent placement and the non-stent placement group

The Kaplan–Meier estimates for 3-, 5-, and 10-year disease-free survival rates in the stent placement group were 97%, 95%, and 81%, respectively, and the rates in the non-stent placement group were 98%, 95%, and 82%, respectively. No significant difference was observed between the two groups (Figure 1).

## Discussion

Duodenal and ampullary lesions exhibit differences; clinically, ampullary lesions are usually more aggressive, grow faster, and are more likely to progress to cancer [11]. Therefore, in most cases, ampullary lesions should be excised, especially when they are large. EP is considered a safe alternative for the complete resection of early ampullary tumors. In this study, 31 patients developed adverse effects post-operatively, including delayed bleeding, pancreatitis, and biliary obstruction. The Kaplan−Meier estimates for disease-free survival rates were not significantly different between the two groups.

The criteria for the curative endoscopic treatment of ampullary tumors remain controversial, and clear diagnostic and therapeutic strategies should be established [12–15]. Before endoscopic resection, CT imaging must be performed to assess the tumor, rather than more invasive biopsies [16]. Endoscopic ultrasound (EUS) is not only a simple diagnostic method, but also can be used for interventional treatment of the biliary and pancreatic diseases [17]. The value of EUS in determining duodenal submucosal invasion has been widely recognized [18–22]. In our study, all patients underwent CT imaging and 82 patients completed the EUS examination. Three patients were excluded owing to pancreatic or bile duct invasion. Standard biopsy forceps might have limitations in the diagnosis of carcinoma as a result of which certain patients might require a second lesion biopsy. Obtaining biopsy specimens before performing endoscopic sphincterotomy is recommended for suspected ampullary lesions, but the agreement with the final histology has been reported to be low (34.4%) [23]. Immunohistochemical tests, K-ras and p53 tests, polymerase chain reaction, and microsatellite instability tests should not be routinely used in biopsies of ampullary tumors to determine the prognosis and/or potential therapeutic response [24]. In our series, the pre-EP biopsy did not identify the foci of adenocarcinoma in 17 patients, which indicates that a negative pre-EP biopsy result is not a reliable parameter for ruling out adenocarcinoma.

The following class of patients were deemed unsuitable for endoscopic therapy: (i) those with biliary pancreatic duct involvement; (ii) those in whom endoscopic resection was impossible due to technical reasons (e.g. diverticulum >4 cm); and (iii) those with incomplete endoscopic resection with positive margins or local recurrence that could not be treated under endoscopy [25, 26]. In this study, the average tumor size was 17 mm, and three patients with biliary and pancreatic duct invasion were excluded. The size of the adenoma and the severity of the patient’s symptoms were associated with the risk of occult malignancy. Most papillary adenomas are diagnosed incidentally [27]; the predominant symptom is abdominal pain. Consistently with previous reports, in our study, 57 patients (53.27%) complained of abdominal pain and their symptoms were relieved after surgery.

A few case series have reported that the combination of epinephrine and methylene blue can minimize bleeding, provide a clearer version of the margins, and reduce complications. However, the need for submucosal injections remains controversial, with little evidence to support its use. In our study, 48 patients received submucosal injection and there was no statistically significant difference in bleeding rates between the non-submucosal and submucosal injection resection groups (data not shown).

The European Society of Gastrointestinal Endoscopy guidelines suggest that non-steroidal anti-inflammatory drugs (NSAIDs) should be used in all patients without contraindication to the administration of these drugs before EP. However, this was a weak recommendation, with low-quality evidence, and hence NSAIDs were not used in this study. In addition, clarity on the timing of stent placement (before or after EP) and the duration of stent retention is lacking. Mostly, the stent was placed after EP, except in two studies [28, 29]. Spadaccini *et al.* [30] reported that the only factor affecting acute pancreatitis as an outcome was same-session prophylactic pancreatic stent placement (odds ratio = 1.72, *P *=* *0.006). Furthermore, in a secondary analysis of randomized–controlled trials, failure of pancreatic stent placement has been reported to exacerbate the risk of pancreatitis after endoscopic retrograde cholangiopancreatography. In our study, none of the patients in the stent or non-stent placement group developed severe pancreatitis. Risk factors for post-EP acute pancreatitis were not associated with age, sex, tumor size, histology, or placement of a pancreatic stent in either univariate or multivariate analysis. These outcomes were consistent with the results of a systematic review of 23 retrospective cohort studies which indicated that there was no statistically significant reduction in the incidence of pancreatitis after papillectomy (odds ratio = 0.71; 95% confidence interval 0.36–1.40; *P *=* *0.325). As bleeding and edema caused by electrocautery may increase the difficulty of stent placement and no patients with severe pancreatitis had been reported previously, pancreatic stents can be placed selectively only in special cases. Furthermore, some studies have pointed out that pancreatic duct stents should be used to prevent pancreatic duct stenosis. However, none of the patients in either group experienced delayed distal pancreatic duct stenosis during follow-up in this study.

This study has certain limitations. First, the study relied solely on medical records owing to its retrospective nature and selection bias could not be eliminated. Second, although the sample size was large, it was not large enough to permit generalization of the results. Therefore, a well-designed prospective randomized–controlled study with a larger sample size should be performed.

Despite these limitations, our study has several strengths. A relatively large-scale database was used and long-term follow-up was done. A previous study reported that 66%−90% of patients were completely cured after EP within a mean follow-up period of 13–36 months (range, 2–174 months) [31]. In our study, the follow-up period was 75 ± 43 months. The management of residual/recurrent adenomas has not yet been standardized. A retrospective study documented successful endoscopic retreatment with Argon plasma coagulation (APC) in 24 patients who had undergone incomplete resection after EP [32]. However, another study did not recommend APC for treating recurrent adenoma [33]. In our study, the cumulative recurrence rate was 12.15%. Most cases of recurrence in the endoscopic treatment group were detected as early-stage mucosal cancer owing to regular endoscopic surveillance and were successfully retreated with additional endoscopic management or subsequent surgery. These findings demonstrate that favorable long-term outcomes could be obtained with EP alone.

In conclusion, our single-center experience with a large cohort of patients confirms that EP provides satisfactory results for treating adenoma of the ampulla of the duodenum.

## Supplementary Material

goad050_Supplementary_Data
