# Supplementary material for: Long-term follow-up of endoscopic papillectomy and the value of preventive pancreatic stent placement (with videos)
Source: Gastroenterol Rep (Oxf). 2023 Oct 18;11:goad050. doi: 10.1093/gastro/goad050 (PMC10585593; doi:10.1093/gastro/goad050)
Supplement: goad050_Supplementary_Data [file goad050_supplementary_data.zip › Supplemetary Table_1.docx]

| **Characteristic** | **Patients** | | | | | | | | | | | | |
| --- | --- | --- | --- | --- | --- | --- | --- | --- | --- | --- | --- | --- | --- |
| No. | #1 | #2 | #3 | #4 | #5 | #6 | #7 | #8 | #9 | #10 | #11 | #12 | #13 |
| Symptom | Abdominal pain | Abdominal pain | Abdominal pain | Physical examination | Abdominal discomfort | Abdominal pain | Abdominal discomfort | Abdominal pain | Abdominal pain | Abdominal pain | Abdominal pain | Abdominal pain | Abdominal pain |
| Age, years | 57 | 44 | 53 | 65 | 62 | 52 | 56 | 48 | 54 | 63 | 51 | 33 | 45 |
| Initial Biopsy | LGD | LGD | LGD | LGD | LGD | LGD | LGD | LGD | HGD | LGD | LGD | HGD | LGD |
| Size, mm | 1.2 | 4 | 1 | 2 | 5 | 1 | 2.5 | 2.4 | 1.5 | 1 | 1.8 | 1.2 | 4 |
| Final pathology | LGD | LGD | LGD | LGD | LGD | LGD | LGD | LGD | LGD | LGD | LGD | HGD | LGD |
| Complications post-endoscopic papillectomy | Pancreatitis | - | - | - | - | - | - | - | - | - | - | - | - |
| Follow-up time, months | 60 | 112 | 52 | 72 | 132 | 96 | 48 | 12 | 154 | 135 | 36 | 53 | 96 |
| Recurrence time, months | 1 | 14 | 8 | 59 | 60 | 3 | 4 | 3 | 7 | 41 | 14 | 19 | 2j'k'k'i |
| Follow-up post recurrence | LGD | LGD | LGD | LGD | LGD | LGD | LGD | LGD | LGD | Adenocarcinoma | LGD | LGD | LGD |

Supplementary Table 1. Characteristics of the thirteen patients with residual or local recurrence of tumor.
